# Identification and degradation characteristics of *Bacillus cereus* strain WD-2 isolated from prochloraz-manganese-contaminated soils

**DOI:** 10.1371/journal.pone.0220975

**Published:** 2019-08-09

**Authors:** Jie Jiang, Meizhen Tang, Junfeng Chen, Yuewei Yang

**Affiliations:** Key Laboratory of Nasihu Lake Wetland Ecosystem & Environment Protection, Qufu Normal University, Qufu, Shandong, China; Universidade de Coimbra, PORTUGAL

## Abstract

The bacterial strain WD-2, which was capable of efficiently degrading prochloraz-manganese, was isolated from soil contaminated with prochloraz-manganese, selected through enrichment culturing and identified as *Bacillus cereus*. Test results indicated that the optimal temperature and pH for bacterial growth were 35–40°C and 7.0–8.0, respectively. The highest degradation rate was above 88–90% when the pH was 7.0~8.0 and reached a maximum value (90.7%) at approximately 8.0. In addition, the bacterium showed the greatest growth ability with an OD_600_ of 0.805 and the highest degradation rate (68.2%) when glucose was chosen as the carbon source, while the difference in nitrogen source had no obvious influence on bacterial growth. The degradation rate exceeded 80% when the NaCl concentration was 0~2% and the rate reached 89.2% at 1%. When the concentration was higher than 7%, the growth of WD-2 and the degradation of prochloraz-manganese were found to be inhibited, and the degradation rate was merely 8.5%. The results indicated that strain WD-2 was able to effectively degrade prochloraz-manganese and might contribute to the bioremediation of contaminated soils.

## Introduction

Prochloraz ([N-propyl-N-2-(2,4,6-trichlorophenoxy)-ethyl]-imidazole-1-carboximide; CAS67747-09-5) is a broad-spectrum fungicide belonging to the class of imidazoles. It was confirmed that the active substance prochloraz and fungicides based on prochloraz are particularly suitable for protecting the wood and wooden materials against attacks or destruction by lignivorous fungi[1,2]. Prochloraz was also used on some cereal crops, fruits and vegetables to control eyespot disease and powdery mildew through the inhibition of ergosterol biosynthesis[3,4]. Prochloraz-manganese is a good alternative to prochloraz due to its similar function and relatively low toxicity and stability[5]. An efficacy test of -prochloraz-manganese indicated that a small amount of the pesticide has significant inhibitory effects on the black spot caused by *Alternaria alternata* and wet bubble disease of white mushroom and could also be used to extend the preservation time of mango and persimmon fruit[6–8]. Therefore, prochloraz-manganese has been widely used in agricultural industries with high dosages since the early 1980s, especially in the mushroom industry[5]. The overuse of prochloraz-manganese is likely to cause considerable pollution problems in soils. As a result, there is an urgent demand to explore an efficient way to solve the problem.

The source of pesticide residue in the environment is mainly agricultural practices through a few routes such as spray drift, surface runoff and field drainage[9]. The fate of residual pesticides was determined by various mechanisms including volatilization, chemical degradation, photodegradation, absorption, hydrolysis, enzymatic degradation and bacterial/fungal degradation[10,11]. The use of microorganisms to degrade pesticides or even macromolecular substances was dependent on their high metabolic diversity and adaptability[12]. In addition, microbial degradation rates were strongly influenced by a wide range of environmental factors such as nutrient availability, the presence of alternative carbon substrates, pH, temperature, and salinity. Therefore, the isolation of a new bacterial strain capable of degrading pesticides and the evaluation of its degradative properties under various laboratory conditions would provide the first step for the practical application of microorganisms in bioremediation. However, due to various environmental restrictions such as low temperature and high alkalinity, the biodegradation efficiency was significantly affected and suppressed.

As a result of the long-term application of prochloraz-manganese, a considerable amount of the pesticide has accumulated in plants, soil and water, which might have some adverse impacts on the environment. Research on environmental toxicology and the metabolism of the residues has been carried out domestically and throughout the world since the early development and application of prochloraz. Chen et al. isolated a Bacillus sp. designated DG-02, which could degrade 95.6% of 50 mg L-13-Phenoxybenzoic acid within 72 h at a pH of 7.7 and 30.9 °C[13]. Liu et al. successfully screened an endophytic quinclorac-degrading bacterial strain (Q3), and the results indicated that the use of this strain to degrade quinclorac for 7 d resulted in a 93% decrease from the initial concentration of 20 mg/L[14]. In the treatment in which glucose was used, degradation percentages of methyl parathion and chlorpyrifos of 98% and 97%, respectively, were obtained in 120 h. This treatment also achieved the highest percent reduction in toxicity when monitored with a high-intensity light source[15]. Moreover, a high-efficiency bacterial strain that was capable of degrading organochlorine pesticides (OCPs) such as aldrin, 4, 4’-DDT, dieldrin, endrin and endrin aldehyde simultaneously was isolated and screened by Belal, E.S.B. et al., while its biodegradation rates ranged between 24.4% and 98%[16]. These results showed that many types of pesticide residues could be effectively degraded by bacteria, even in extreme environments. However, there have been few studies on the bacteria that could effectively degrade prochloraz-manganese. Because of the slow metabolic degradation of prochloraz-manganese in the environment, a bacterial strain that could efficiently degrade the pesticide is now urgently needed and is the focus of this study.

The goal of this study is to isolate and characterize a bacterial strain capable of degrading prochloraz-manganese. Furthermore, the growth and degradation characteristics of this strain were also investigated under various conditions. The research results enrich the prochloraz-manganese-degrading microbial species resource and provide a valuable technical reference for the biodegradation of this pesticide.

## Materials and methods

### Soil collection

Soil samples (obtained from the top 20 cm of soils) were taken from a prochloraz-manganese-contaminated field in Tianjin, China(117.123,39.24). After drying, pulverizing, and sieving the soil with a 2 mm sieve, 100 g of sample soil was taken, and the strain was isolated in quadruplicate. The study was carried out on private land, and we confirm that the owner of the land gave permission to conduct the study on this site and the field studies did not involve endangered or protected species.

### Bacterial isolation and medium

Analytical-grade prochloraz-manganese (98% purity) was obtained from the Kangbaotai Fine Chemical Company (Hubei, China).

Soil samples (5.0 g) were added into sterilized triangular flasks (250 mL), which contained 100 mL of mineral salt medium (MSM) and 5.0 mg/mL of prochloraz-manganese. The bacterial suspension was cultivated at 37°C with a rotation speed of 150 rpm and transferred into fresh MSM every 3 d for continuous enrichment with 5% inoculum. After 4 transfers, the concentration of prochloraz-manganese in the medium was gradually increased to 500 mg/L, and the suspension was then cultivated for 3 d under the same conditions as described above. A total of 0.1 mL of the suspension was taken and spread on beef cream peptone medium, GAUZEˊs medium No .1 and potato medium (for the cultivation of bacteria, fungi, and actinomycetes, respectively) and cultivated for 3 d. Several well-grown strains were selected, isolated and purified. The purified strains were transferred to the MSM media (containing 500 mg/mL of prochloraz-manganese) to determine their degradation ability. Then, the strain with the best degradation ability was selected and stored at 4 °C.

### Characterization of the bacteria

#### The morphological characteristics of the isolated bacteria

The preserved degrading strains were inoculated on the solid medium by the spread plate method and cultured at 37°C for 2 d. The colony characteristics and the morphological structure of the cells were observed under a Nikon 80i microscope. Then, gram staining was performed on the strain capable of degradation for physiological and biochemical identification.

#### 16S rDNA sequence analysis

DNA extraction of the isolated strain was completed by the Cetyltrimethylammonium bromide (CTAB) method. The 16S rDNA amplification procedure and phylogenetic analyses were performed as described by Weisburg W G et al.[17]. 16S rDNA primers were used to conduct PCR amplification using the DNA extracted from the isolated strain as a template. The forward primer for the bacterium was 8f (5’-AGAGTTTGATCCTGGCTCAG-3’, 20 bp), and the reverse primer was 1492r (5’-GGTTACCTTGTTACGACTT-3’, 19 bp). PCR products were transported to Shanghai Sangon Biological Engineering Technology and Services Co. Ltd. (Shanghai, China) for sequencing. The sequences were analysed using the BLAST program (Altschul et al., 1990) of the National Center for Biotechnology Information (NCBI) and constructed with the MEGA 6.0 software package and neighbour-joining algorithm. The evolutionary distances were computed using the Kimura 2-parameter method[18–20]. Bootstrap analysis was performed by means of 1000 replicates[21].

### Growth curve of the isolated bacteria

A 0.5 mL aliquot of bacterial suspension was added to a sterilized conical flask containing 100 mL of LBM and incubated in a shaking incubator at different temperatures (10°C, 18°C, 28 °C and 37 °C) at 150 rpm. The cell density was measured at 600 nm (OD_600_) at intervals of 5 h. The optimal culture time of the degraded strains at different temperatures was obtained, and the growth curve of the strains was drawn.

### Effects of different culture conditions on bacterial growth and the degradation rate of prochloraz-manganese

The strain was cultivated to a logarithmic stage in Luria-Bertani (LB) media and then inoculated (by a 5% inoculation amount) into the inorganic salt medium with a high prochloraz-manganese concentration of 500 mg/L. After incubation at a speed of 150 rpm under various culture conditions, samples were separately collected to determine the OD_600_ and the degradation rate of prochloraz-manganese of each culture medium.

The medium was cultured at 10 °C, 18 °C, 28 °C and 37 °C to assess the effect of temperature. Glucose, sucrose, maltose, and soluble starch (concentration of 1 g/L) were added, and the medium was cultured at 37 °C to assess the effect of the carbon source. A concentration of 1 g/L of nitrate, urea, ammonium chloride, peptone was added individually to cultures at 28 °C to assess the effect of the nitrogen source. The pH of the culture solution was 4.0, 5.0, 6.0, 7.0, 8.0, 9.0, or 10.0 respectively, and culture was performed at 28 °C. The initial salinity of the culture solution was set to 0%, 1%, 3%, 5%, 7%, and 9% respectively, cultured at 28 °C. The inorganic salt media were prepared separately with different prochloraz-manganese concentrations of 100 mg/L, 200 mg/L, 300 mg/L, 500 mg/L, 750 mg/L and 1000 mg/L respectively, cultured at 37 °C. Each treatment was repeated 3 times.

### Detection of prochloraz-manganese

One millilitre of the liquid medium sample was mixed with 1 mL of dichloromethane as well as 1 mL of 2.0% sodium chloride solution. After thirty-minute oscillation extraction and stratification, the combined organic phases from the substratum were dried with anhydrous Na_2_SO_4_ and then evaporated using a rotary vacuum evaporator. The mixtures were dissolved in methanol to a final volume of 5.0 mL. Waters e2695 high performance liquid chromatography (which is equipped with a Waters 2489 UV detector and Empower3 data processing system); Chromatographic column: Agilent ZORBAX Extend-C18, 250 mmX x 4.6 mm (i.d.), 5 μm. Methanol/water = 9/1(v/ v, adjust pH to 3.0 with H_3_PO_4_) was used in the mobile phase, and the flow rate was 0.8m·min^-1^. The column temperature was 30°C. The detection wavelength was 210 nm. Sample quantity was 20μL. Retention time (Rt) was 5.384 min, and its detection limit ranged from 0.24 to 10.8 mg/kg. The limits of detection (LOD) and limits of quantification (LOQ) were calculated on the basis of signal-to-noise ratios (S/N) of 3 and 10, respectively. The LOD and LOQ values were 0.036 and 0.120mg/kg. The recovery of prochloraz-manganese was in the range of 87.6–108.2%, with a relative standard deviation of 2–13%.

## Results and analyses

### Identification of the prochloraz-manganese-degrading strain

**Fig 1:**
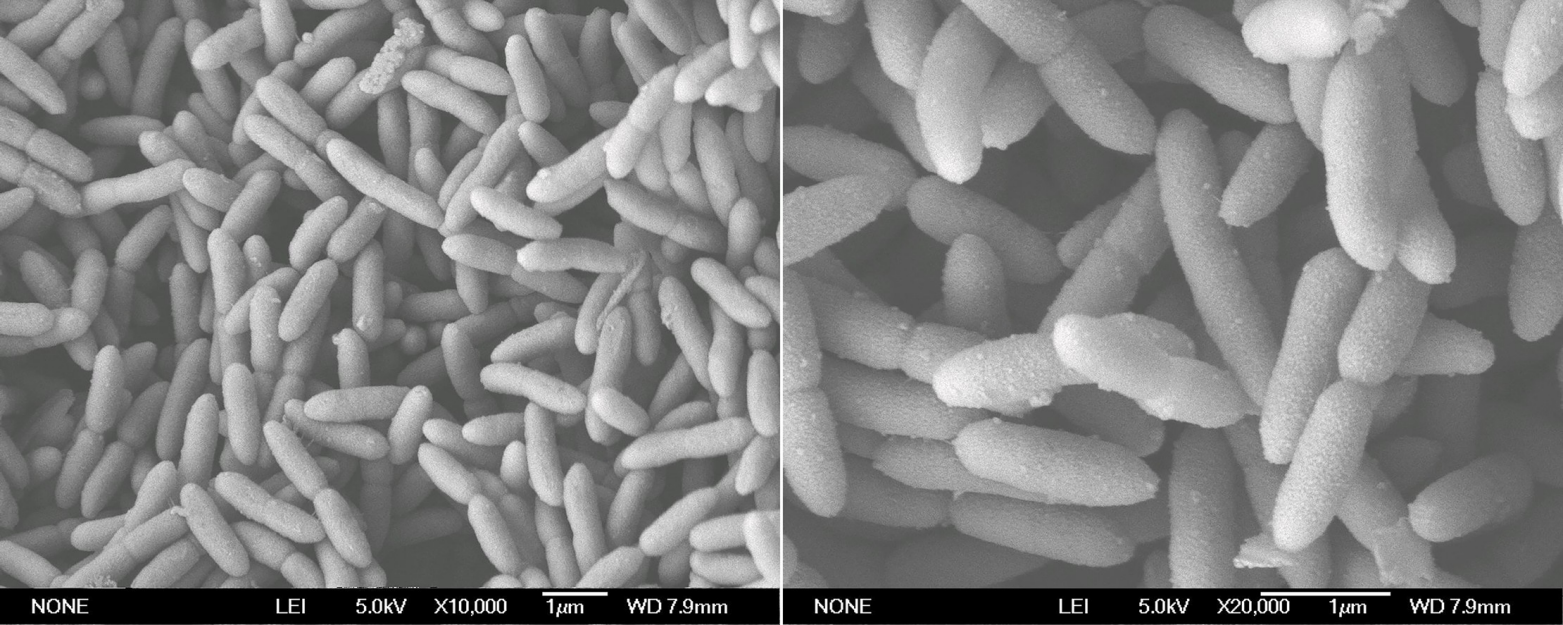
SEM image of strain WD-2.

Through repeated separation and purification, the strain with the highest prochloraz-manganese degradation rate, designated WD-2, was isolated from the pesticide-contaminated soil. After 24 h of culture in the MSM medium, the colonial morphology was as follows: the bacteria were white, moist and circular and had a smooth, convex surface as well as a neat edge. SEM images of strain WD-2 showed a short rod shape with a size of (0.6–0.8) μm × (1.1–1.4) μm in Fig 1. Biochemical characteristics assayed for the strain WD-2 are shown in Table 1.

**Table 1:** Biochemical characteristics of the strain WD-2.

biochemical test	result	biochemical test	result
Gram staining	+^※^	Methyl red	-
Gelatine hydrolysis	-	Ox/F glucose	+
Oxidase	-	Indole	+
Catalase	+	Voges-Proskauer	+
Amylolytic enzyme	+	Citrate	+

^※^+: positive; -: negative.

**Fig 2:**
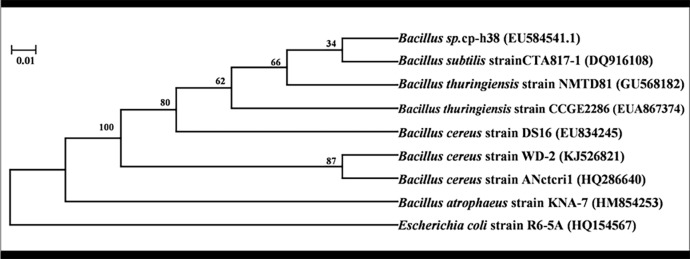
The 16S rDNA phylogenetic tree of strain WD-2.

According to the results from Shanghai Biological Engineering Co., Ltd., the length of the specific 16S rDNA sequence of strain WD-2 was 1250bp (GenBank accession number KJ526821). A BLAST analysis of the bacterial strain WD-2 was performed, and the results are shown in Table 2. The phylogenetic tree was constructed by MEGA 6.0 software. The phylogenetic tree of WD-2 is shown in Fig 2. The results indicated that the sequence of the 16S rDNA gene of strain WD-2 showed a high sequence similarity (more than 99%) to the genus *Bacillus*. Based on the physiological and biochemical characteristics and the 16S rDNA of the strain, strain WD-2 could be preliminarily identified as *Bacillus cereus*.

**Table 2:** BLAST analysis of the bacterial strain WD-2.

strains	accession	length	homology
*Bacillus cereus*; NS 58	AJ577274	1382 bp	0.992
*Bacillus cereus*; LKT 1/2	AJ577284	1377 bp	0.992
*Bacillus cereus*; F 3371/93	AJ577287	1386 bp	0.992
*Bacillus cereus*; ATCC 4342	AJ577288	1430 bp	0.992
*Bacillus cereus*; ATCC 10987	AJ577290	1372 bp	0.992
*Bacillus cereus*; LKT 1/1	AJ577278	1371 bp	0.992
*Bacillus cereus*; OH 599	AJ577286	1371 bp	0.992
*Bacillus cereus*; B 319	AJ577285	1371 bp	0.992
*Bacillus cereus*; RIVM BC00068	AJ577283	1437 bp	0.992
*Bacillus cereus*; G9241	AY425946	1466 bp	0.992

The degradation of pesticides by the genus *Bacillus* has been widely reported by previous studies. Tang et al. isolated a *Bacillus sp*. (TAP-1) strain capable of degrading triazophos, and Ali M et al. isolated a *Bacillus pumilus* strain (WI) capable of degrading organophosphate. In addition, Zhang et al. successfully isolated *Bacillus cereus* strain Y1, which could efficiently decompose deltamethrin[22–24]. These results indicated that the genus *Bacillus* plays an essential role in the biodegradation of pesticides.

### Growth curves of the strain WD-2

**Fig 3:**
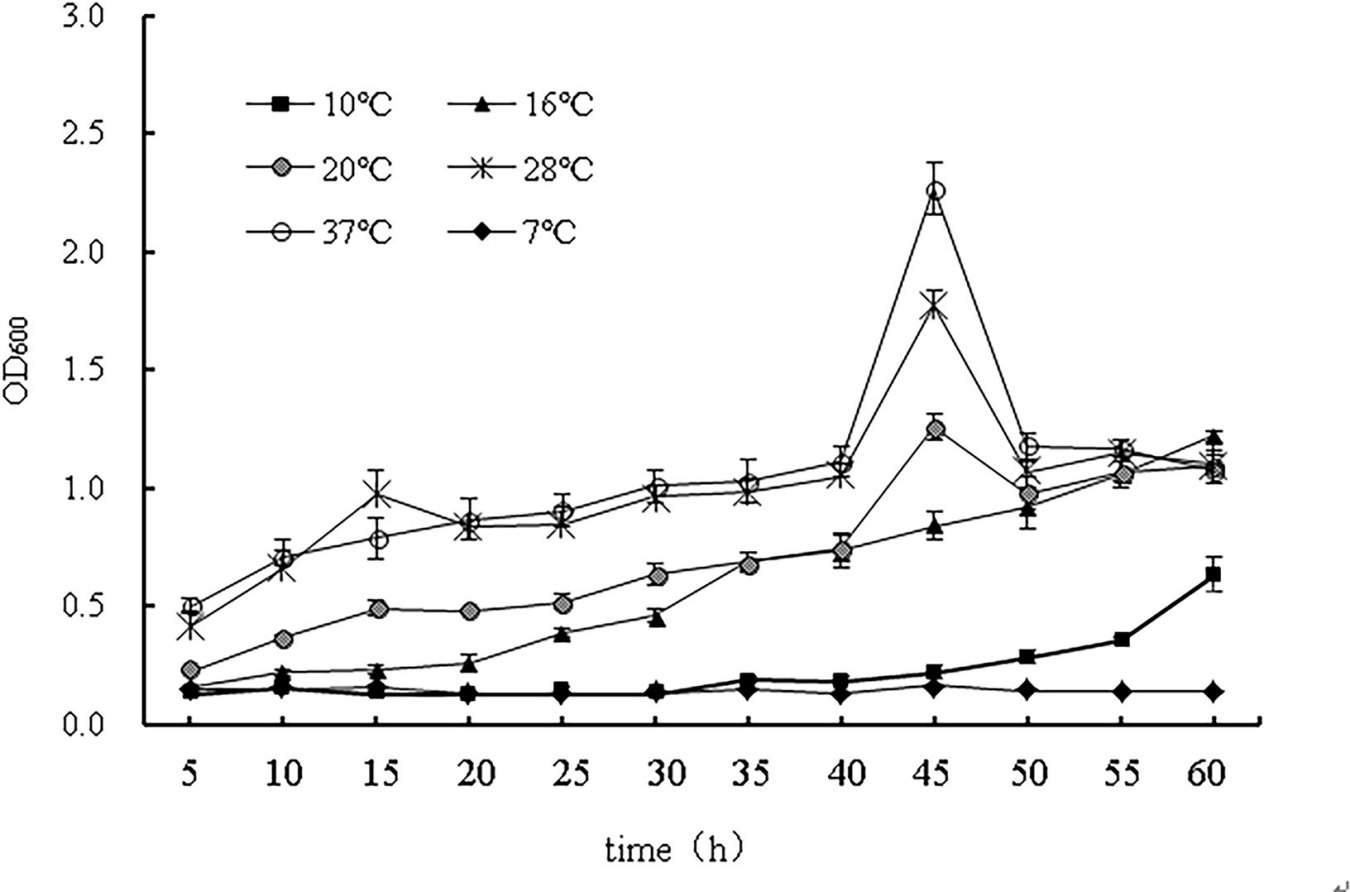
Growth curves of strain WD-2 at different culture temperatures.

Growth curves of strain WD-2 at different culture temperatures are shown in Fig 3. When the culture temperature was between 20°C and 37°C, the OD_600_ increased with increasing temperature to a maximum at 45 h. When the culture temperature was above 16°C, strain WD-2 grew slowly and could barely grow at a low temperature of 10 °C. The results showed that the optimal temperature for strain WD-2 growth was 37°C. When the culture temperature was approximately 37°C, the lag, logarithmic, stationary and death phases of the strain were from 15 to 40 h, 40 to 45 h, 45 to 50 h, and 50 h and later, respectively.

### Effects of different culture conditions

#### Effects of temperature

**Fig 4:**
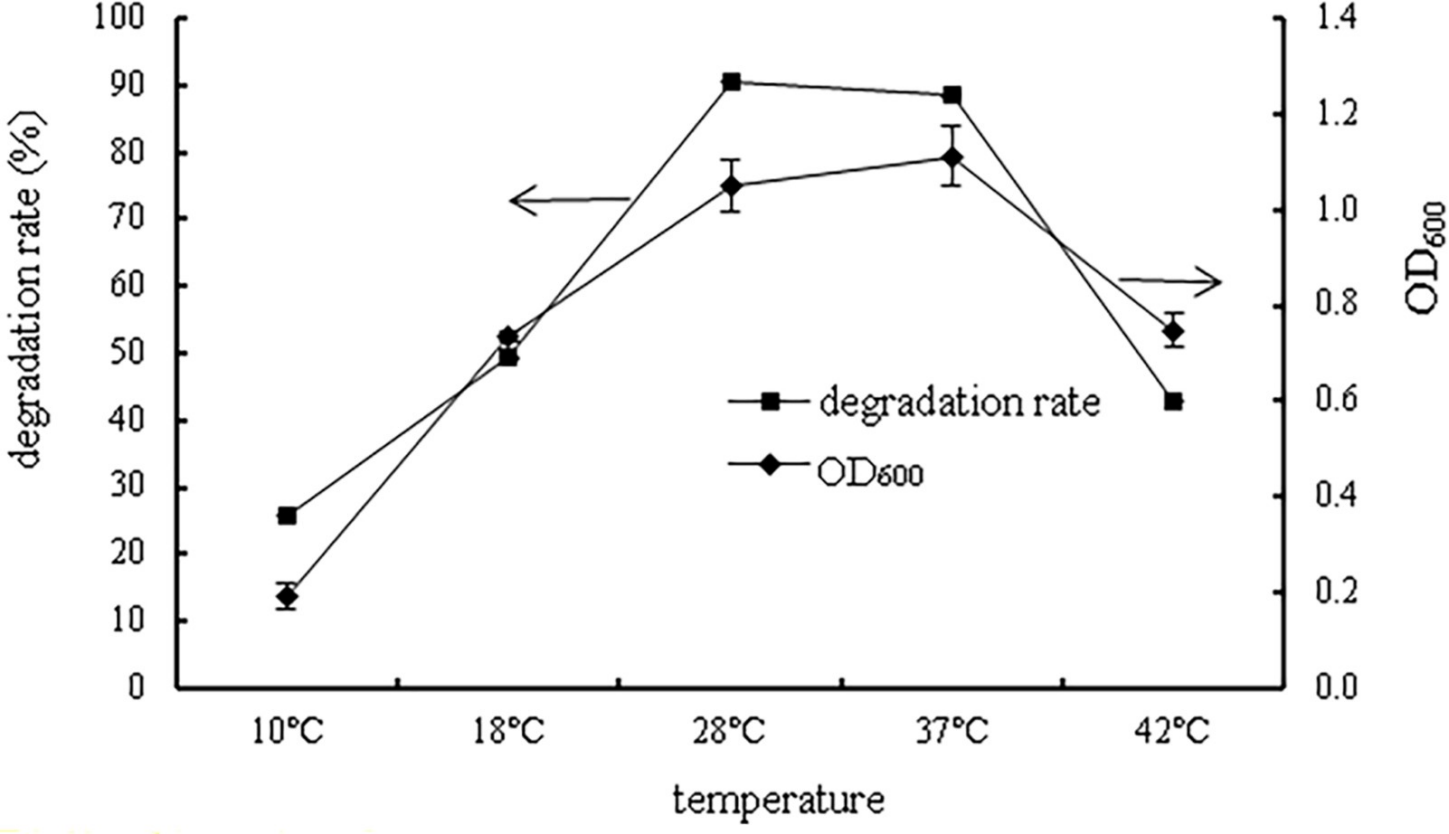
The effect of temperature on bacterial growth and the degradation rate of prochloraz-manganese.

The effect of temperature on bacterial growth and the degradation rate of prochloraz-manganese are represented in Fig 4. Both the degradation rate and bacterial growth significantly increased at 10–37°C and decreased after 37 °C, indicating that the degradative ability of WD-2 was positively correlated with the OD_600。_ When the temperature was 37 °C, both the growth and the degradation rate of strain WD-2 reached their maximum, and the degradation rate of prochloraz-manganese reached 90.6%. In addition, when the temperature was approximately 18–42°C, the strains were able to grow well and significantly degrade prochloraz-manganese. This result indicated that the strain WD-2 could survive under a wide range of temperatures, which will enable the strain to work effectively in practical applications.

#### Effects of carbon sources

**Fig 5:**
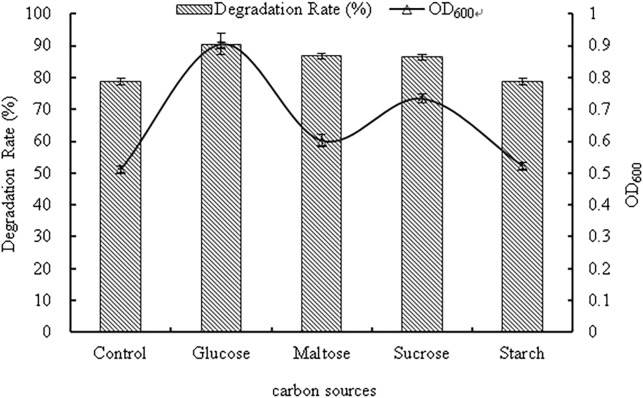
The effect of carbon sources on bacterial growth and the degradation rate of prochloraz-manganese.

The effects of different carbon sources on strain WD-2 are depicted in Fig 5. When the carbon sources differed, the growth of WD-2 and the degradation rate of prochloraz-manganese were positively correlated, and the effect of different carbon sources was glucose>sucrose>maltose>starch. When glucose was used as the carbon source, the growth of strain WD-2 was maximal, and the OD_600_ value reached 0.905. The degradation rate of prochloraz-manganese increased by 11.5% compared with that of the control group, reaching 90.2%. In addition, when starch was chosen as the carbon source, the growth and the degradation ability of the strain were limited. The reason for this result may be that glucose is a small molecular substance that is easily utilized by the strain WD-2, and starch is not easily utilized since it is a macromolecular substance. Based on these results, glucose was used as the optimum carbon source in further experiments. Malghani et al. reported that a bacterial strain capable of degrading profenofos preferentially utilized glucose as a carbon source in the medium. The degradation rate of prochloraz-manganese increased by 8.0% when sucrose or maltose was used as the carbon source[25].

#### Effects of nitrogen sources

**Fig 6:**
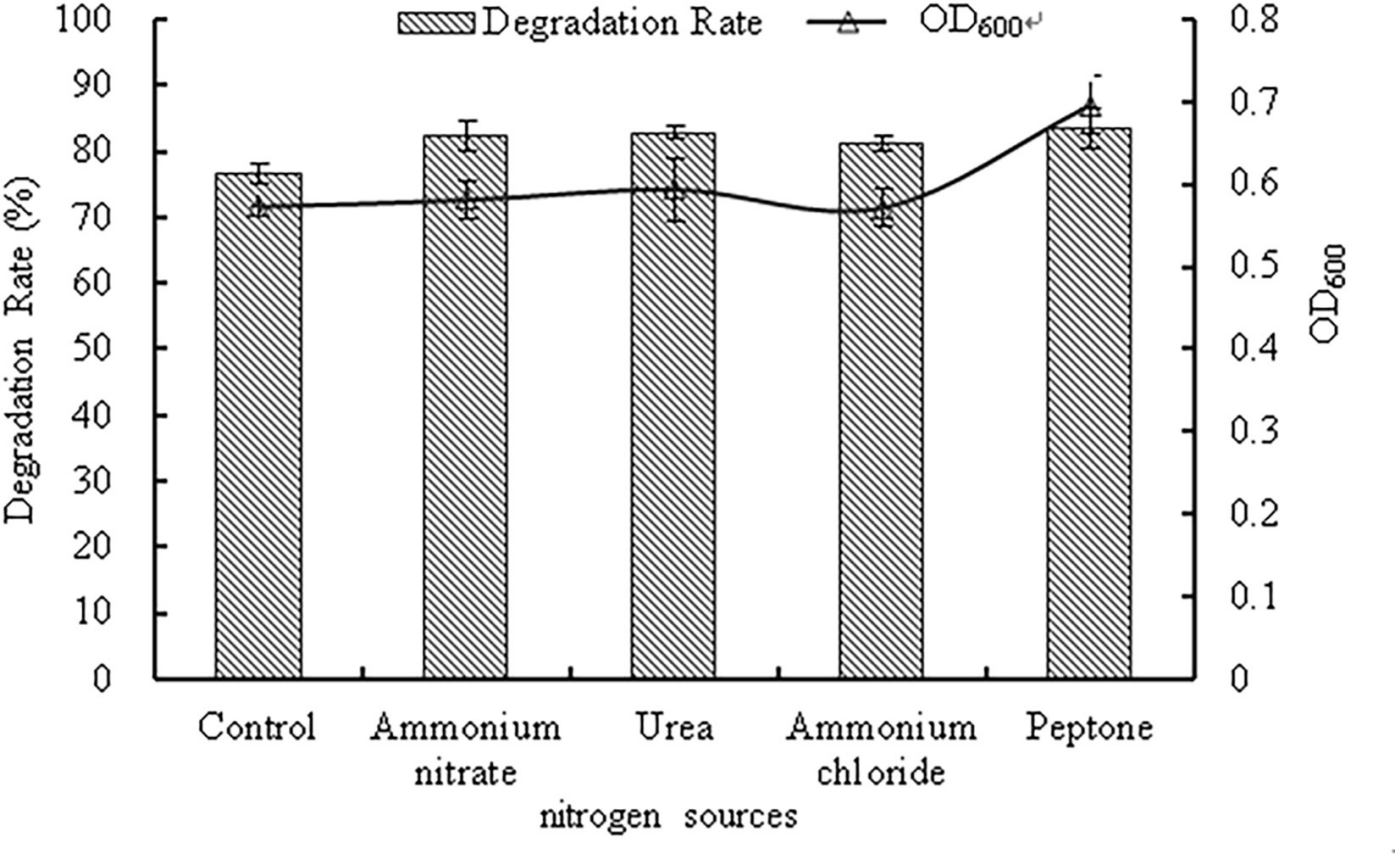
The effect of nitrogen sources on bacterial growth and the degradation rate of prochloraz-manganese.

The effects of different nitrogen sources on strain WD-2 are depicted in Fig 6. The OD_600_ value of strain WD-2 was between 0.83–0.86 under different nitrogen sources (peptone, ammonium chloride, ammonium nitrate and urea), and the degradation rate of prochloraz-manganese was 81.2–83.5%. Compared with the control group, the experimental groups showed no notable difference in the growth of the strain and the degradation rate of the prochloraz-manganese. The results indicated that the nitrogen source had no significant impact on the growth of strain WD-2 and its ability to degrade prochloraz-manganese. The reason for the analysis may be that the strain WD-2 mainly uses carbon sources as nutrients, and the utilization rate of nitrogen sources is low. Considering the cost of practical application, urea was chosen as the nitrogen source in further studies.

#### Effects of pH

**Fig 7:**
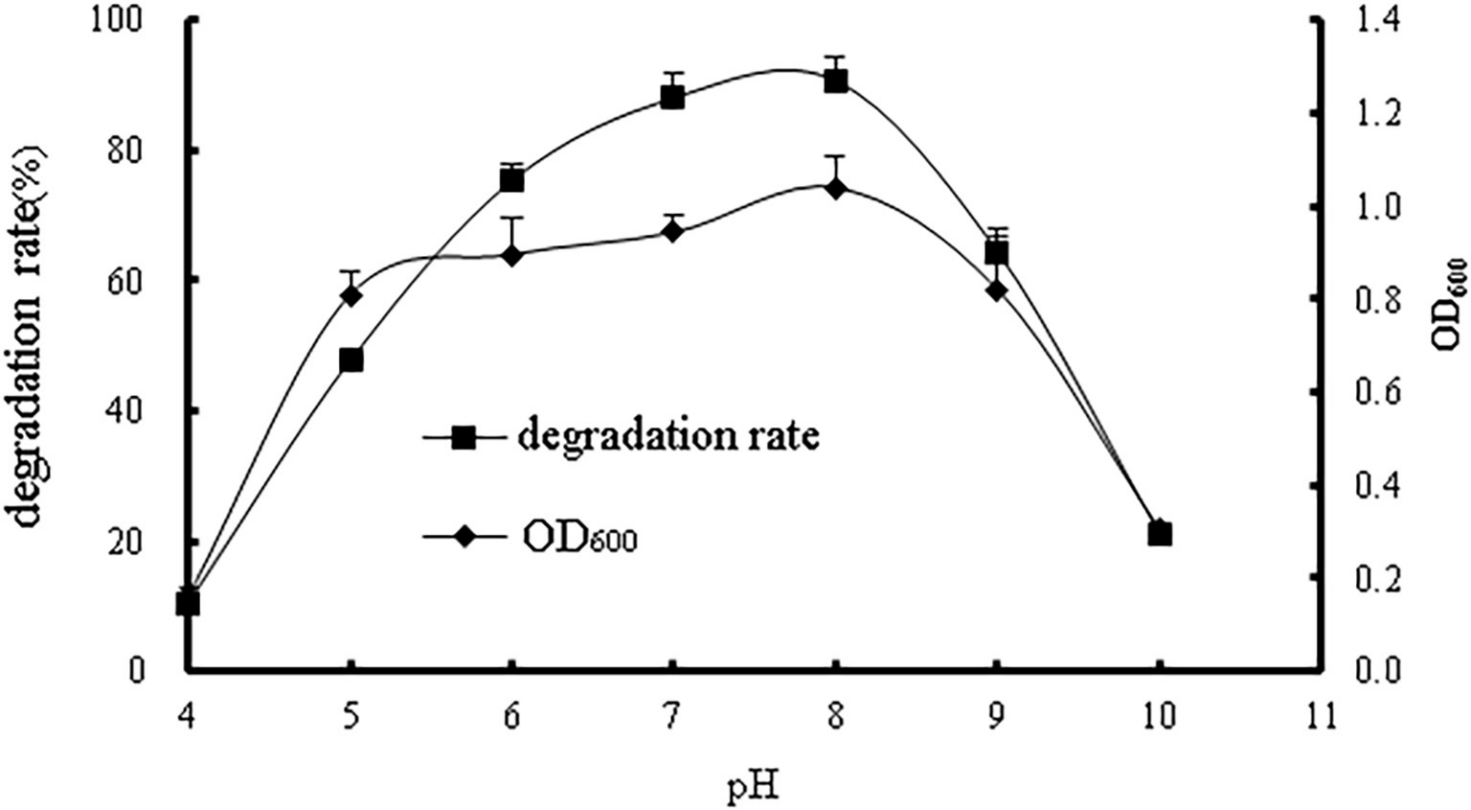
The effect of pH values on bacterial growth and the degradation rate of prochloraz-manganese.

The effects of different pH values on strain WD-2 are depicted in Fig 7. The growth rate of strain WD-2 and the degradation rate of prochloraz-manganese increased in the pH range of 4.0~8.0. The degradation rate reached more than 88% in the pH range of 7.0~8.0 and reached a maximum of 90.7% at a pH of 8.0. When the pH was greater than 10.0 or less than 4.0, the prochloraz-manganese could barely be degraded. When the pH was above 8.0, the degradation efficiency began to decrease.

The effects of pH conditions on bacterial growth and degradation activity have been reported in previous studies. For example, Zhao et al. reported that slightly acidic conditions were favourable for the growth of the strain ZHJ6 and its degradation of methamidophos. Singh D P et al. reported that the optimal pH for the growth of the strain PUPCCC 64 and its degradation of chlorpyrifos was 7.0[26,27].

According to the results, strain WD-2 had the best degradation under weakly alkaline conditions, and the optimum pH for degradation was 7.0–8.0. WD-2 was more suitable for repairing the pollution of weak alkaline environments in practical applications and should be controlled within a certain pH range.

#### Effects of salinity

**Fig 8:**
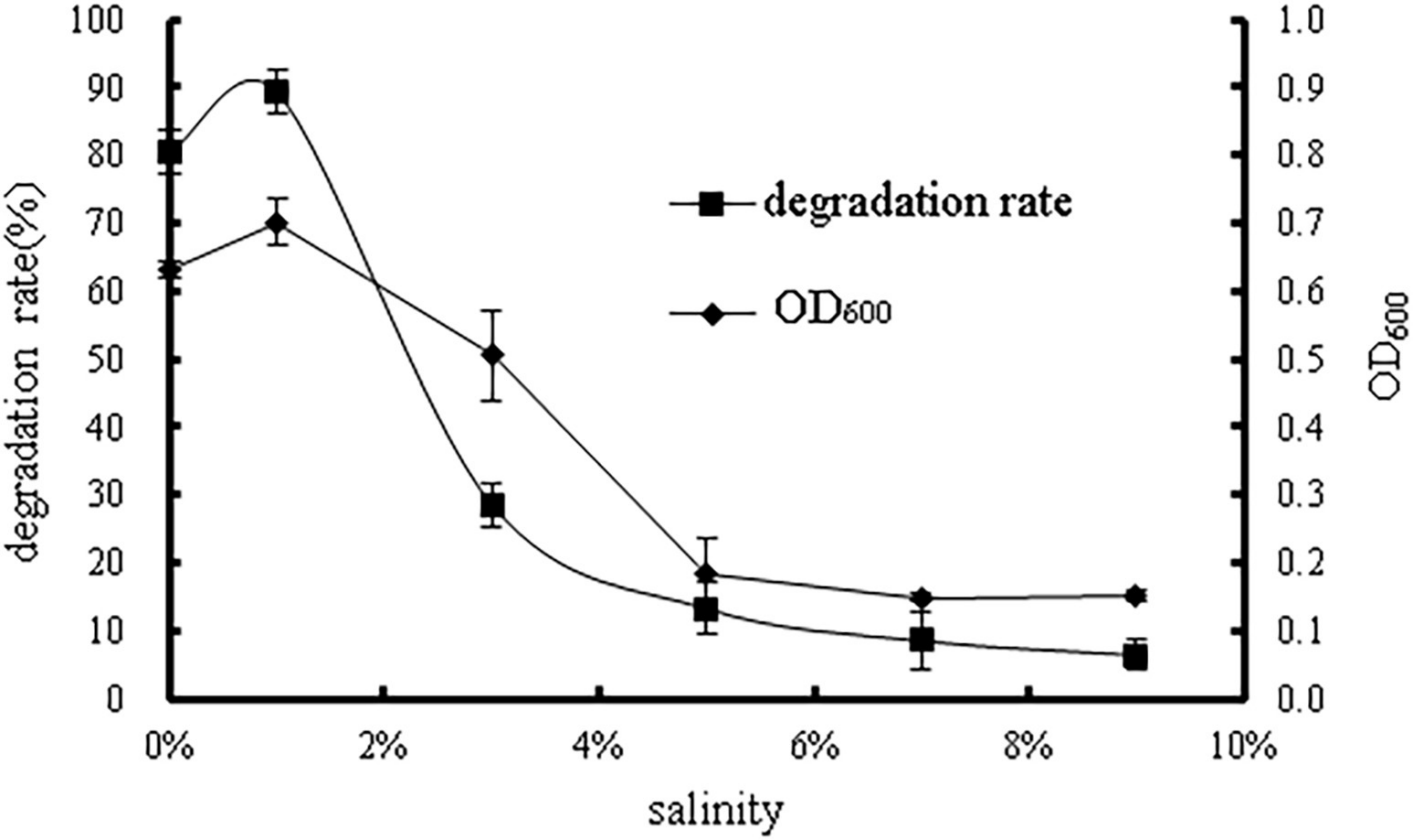
The effect of salinity on bacterial growth and the degradation rate of prochloraz-manganese.

Salinity may have considerable effects on biomass and microbial species, microbial physiological changes, and microbial molecules and cells. As shown in Fig 8, the bacterial growth,as well as degradation rate, decreased with salinity varying from 1% to 9%. The growth inhibition at high salinity may have been caused by the extreme dehydration of the cells of strain WD-2. Although the optimal salinity was observed at 1%, there was a slight difference in bacterial growth and degradation rate at salinities between 0% and 1%, indicating that the strain WD-2 may have a certain degree of tolerance to low salinity. A. Aziz et al. isolated three benzo[a]pyrene-degrading bacterial strains from sea sediments, and approximately 41% of an initial 50 mg/L benzo[a]pyrene concentration was successfully decomposed by the bacterial consortium after 8 days of incubation in a simulated seawater environment (28 ppm of NaCl)[28]. These results provide theoretical support for the application of microorganisms under different salinity conditions. In conclusion, the optimum salinity for growth of WD-2 was identified as 1%. Under low salinity conditions, the strain grew well and was highly effective at degrading prochloraz-manganese.

### Biodegradation kinetics

By studying the kinetics equations of the degradation process, the removal of contaminants could be predicted, which is necessary for the modification of the treatment method as well as the optimization of the biochemical disposal process.

**Fig 9:**
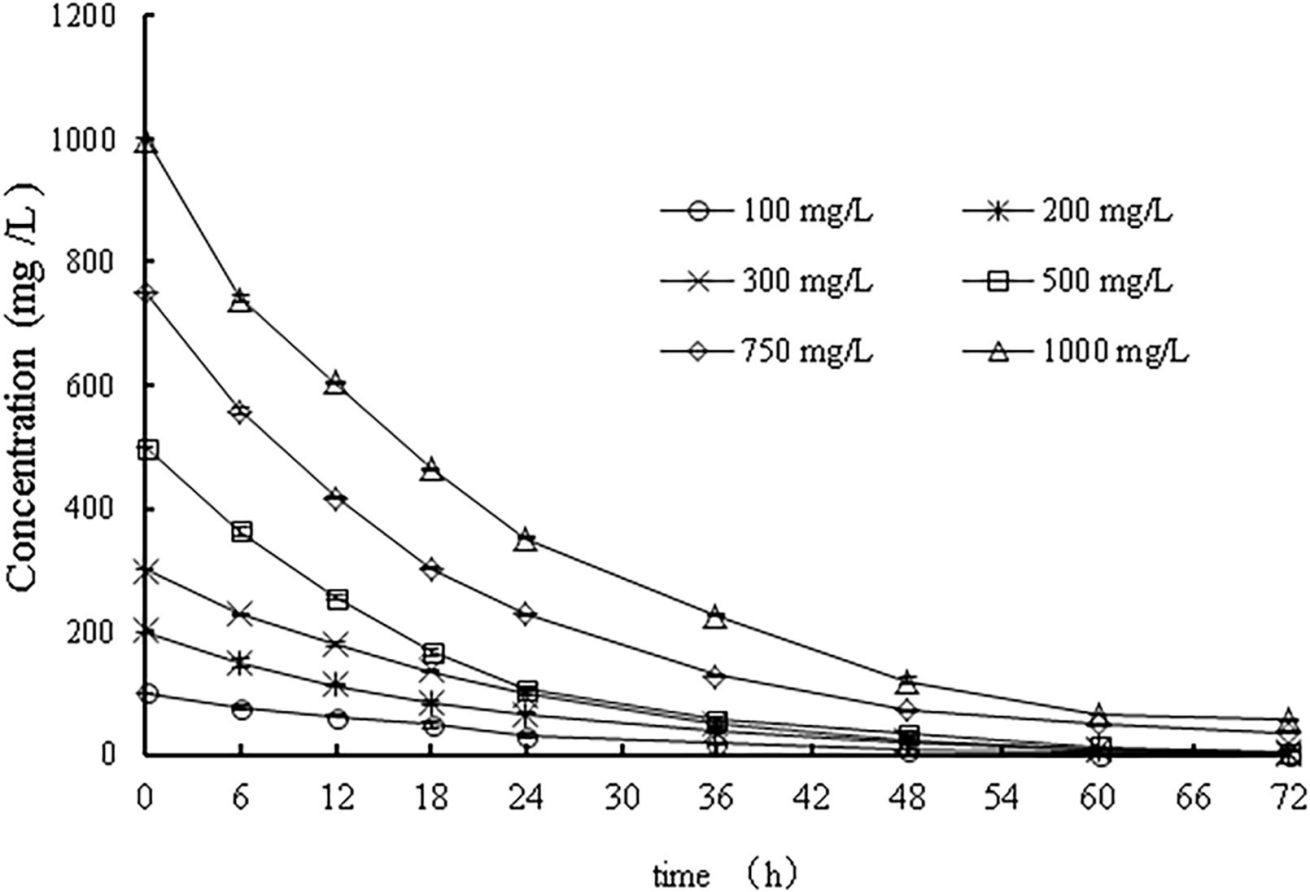
The curve of prochloraz-manganese degradation by strain WD-2 at different initial concentrations.

The first-order kinetic model has been widely applied for the prediction of bacterial treatment results. The prochloraz-manganese concentrations were measured, and time curves of the concentrations of prochloraz-manganese were drawn, as shown in Fig 9. It was assumed that strain WD-2 follows the first-order reaction kinetics equation for prochloraz-manganese biodegradation, and the kinetic equation was as follows:

ln(C/mg/L)=−kt+A (1)

In formula (1), k—kinetic constant of the prochloraz-manganese degradation rate; C—the concentration of prochloraz-manganese (value C/mg/L); t—biodegradation time, h; and A—constant.

Based on the results shown in Fig 9, the kinetic equation can be obtained by applying the formula (Table 3). When the initial concentration of prochloraz-manganese was less than 500 mg/L, the half-life of WD-2 degradation of prochloraz-manganese was similar, which corresponds to the degradation kinetics equation lnC = −0.061t + A, and the half-life was 11.06 h. The correlation coefficients (r) of each k reached 0.9830, 0.9850, 0.9945, 0.9976, 0.9956 and 0.9954. When the initial concentration became higher than 500 mg/L, the degradation rate constant decreased with the initial concentration of prochloraz-manganese, indicating that a high concentration of prochloraz-manganese might inhibit the strain’s degradation ability of the strain. Furthermore, the degree of influence may also increase with the initial concentration of prochloraz-manganese.

**Table 3:** First-order kinetics model and half-time in different initial concentrations.

initial concentration (mg/L)	kinetics equation	half-time (h)	correlation coefficient (r)
100	ln*C* = -0.067X+137.99	10.35	0.9830
200	Ln*C* = -0.061X+246.36	11.36	0.9850
300	ln*C* = -0.061X+367.35	11.36	0.9945
500	ln*C* = -0.062X+519.04	11.18	0.9976
750	ln*C* = -0.044X+685.23	15.75	0.9956
1000	ln*C* = -0.042X+973.5	16.5	0.9954

## Conclusions

1. The strain with high degradation activity was isolated from soils contaminated with prochloraz-manganese by enrichment culture. The strain was preliminarily identified as *Bacillus cereus* and named *Bacillus cereus* WD-2 (GenBank accession number JX114949).
2. The optimal carbon source, temperature, pH, and salinity scope for the growth of WD-2 were glucose, 35–40°C, 7.0–8.0, and within 1%, respectively. When glucose was chosen as the carbon source, the growth ability (OD_600_ value of 0.905) and degrading abilities (90.2%) both reached a maximum. However, the difference in nitrogen sources seemed to have little influence on the growth of WD-2. Based on the study of the growth characteristics of WD-2, it can be concluded that strain WD-2 is adapted to a wide range of temperature and pH conditions. The kinetics study indicated that the degradative ability of the strain was negatively correlated with the initial concentration of prochloraz-manganese. Thus, the strain has a substantial degree of potential application for the bioremediation of soils contaminated by prochloraz-manganese, especially soils with low alkalinity and salinity.
